# Development of an Interactive Touchless Technology Based on Static-Electricity-Induced Luminescence

**DOI:** 10.3390/s23052462

**Published:** 2023-02-23

**Authors:** Keina Abe, Taiga Eguchi, Tatsuya Oyama, Yuki Fujio, Kazuya Kikunaga

**Affiliations:** 1 Faculty of Science and Engineering, Saga University, 1-Honjo, Saga 840-8502, Japan; 2 Sensing System Research Center, National Institute of Advanced Industrial Science and Technology, 807-1 Shuku-Machi, Tosu, Saga 841-0052, Japan

**Keywords:** touchless, finger, luminescence, static electricity

## Abstract

Touchless technology has garnered significant interest in recent years because of its effectiveness in combating infectious diseases such as the novel coronavirus (COVID-19). The goal of this study was to develop an inexpensive and high-precision touchless technology. A base substrate was coated with a luminescent material that emitted static-electricity-induced luminescence (SEL), and it was applied at high voltage. An inexpensive web camera was used to verify the relationship between the non-contact distance to a needle and the applied-voltage-triggered luminescence. The SEL was emitted at 20–200 mm from the luminescent device upon voltage application, and the web camera detected the SEL position with an accuracy of less than 1 mm. We used this developed touchless technology to demonstrate a highly accurate real-time detection of the position of a human finger based on SEL.

## 1. Introduction

The coronavirus (COVID-19) pandemic began in 2020 and has now spread worldwide. Although vaccines have been developed to curb the spread of the disease, outbreaks of infectious variants continue to diminish the utility of available vaccines and drugs [1]. An infection presumably takes several years to subside. Therefore, worldwide efforts are being made to maintain economic activities by easing movement restrictions while preventing further infections, rather than absolutely avoiding the spread of infection through lockdowns. However, easing movement restrictions does not eliminate the possibility of infection, even though such measures may enable the general population to return to a new ‘normal’ life. Therefore, providing individuals with a sense of security to facilitate their activities is important.

Contactless technology, which enables device operation without human contact, is gaining attention for reducing the possibility of infections and facilitating people to continue their daily activities [2,3]. This technology ensures hygiene and prevents the spread of infection in places with an unspecified number of people, such as shopping malls, public facilities, and hospitals. In recent years, infrared sensors that enable touchless control in passenger elevators [4], Internet of Things devices that enable voice controlled on/off operation of switches [5], touchless pointing devices based on three-dimensional motion sensors [6], and motion-sensor-controlled holographic displays [7] have been developed and widely implemented in various real-life situations, particularly during the COVID-19 pandemic. Further, the development of input interfaces for movement- or voice-controlled electrical devices is gaining traction.

In this study, we developed a new touchless technology using a thin luminescent device formed by combining electrostatic and luminescent materials. The device was developed for application in towns, companies, factories, construction sites, etc., with an unspecified number of people. We focused on the construction of a simple and practical device that detects complex finger movements and can be easily installed in existing locations. To ensure that the device exhibits homology with complex movements as well as instils confidence among the users, the device needs to function effectively at larger finger-to-device distances and detect the finger’s spatial position with a high accuracy. Table 1 lists the principles utilised in existing touchless input interfaces and their characteristics. Capacitive touch panels [8] are compatible with complex movements (high accuracy in detecting positions) and can be easily installed, and thus, touchless panels based on this principle have been widely developed [9]. However, the capacitive principle is not suitable for non-proximity applications, and to bridge this gap, infrared-based technologies [10,11] have been developed, including ultrasound [12], image recognition, and artificial-intelligence-based cameras [13]. Infrared sensors such as an oncoming camera must be installed in an existing system, e.g., a wall. In the case of ultrasound devices, which detect sound waves reflected from a finger, the phase difference has to be measured over a larger area, as the detection position moves further away from the finger. Hence, a large camera is required to increase the detection accuracy of the X and Y coordinates. There are two types of devices based on the principle of camera depth: the first type (an opposed type) is installed on the opposite side of the finger, i.e., deeper than the wall, and the other type is installed at the top (or bottom) using two cameras at certain angles. The former device type requires the construction of walls, whereas the latter detects planar positions with poor accuracy, which can be improved by increasing the angle. However, this method of realising non-proximal detection results in significant inaccuracies in the detected position. To increase the position-detection accuracy, a highly sophisticated image recognition method along with a large-sized sensing device are required. An advanced image recognition can be realised by preparing a large number of images for learning and using a high-performance computer to process these images, although developing the requisite system as well as its installation for such an implementation are significant challenges. In addition, the corresponding sensor should be able to operate at a non-proximal distance from the finger and detect the input position with high accuracy. The device developed in this study is based on the principle of static-electricity-induced luminescence (SEL) [14] discovered by Kikunaga et al. The ultimate aim of this study was to develop a new touchless technology using a thin luminescent device that can overcome the aforementioned challenges associated with non-proximity, accuracy, and installation.

## 2. Methods

### 2.1. Luminescent Devices and Detection

Thin luminescent devices (film thickness ~50 μm) were screen-printed with epoxy resin and SrAl_2_O_4_:Eu^2+^ (luminescent material) [14,15] on aluminium foil or paper as base substrates. The epoxy resin serves as a protective coating. To detect the luminescence, we developed an image processing software that processed every five frames of the input image obtained from a web camera operating at a speed of 30 fps. The processing software was developed using C++ and an image-processing library called OpenCV (Intel. ver. 4.5.2). SEL arises from Eu^2+^ and has a broad emission spectrum, mainly around 510 nm [14,15,16]. However, because an RGB (where R, G, and B denote red, green, and blue, respectively) image is typically used as the input [17,18], evaluating minute changes in the hue is difficult. Consequently, in our case, the input image was converted from the RGB colour space to the HSV (represented by hue, saturation, and value, respectively) colour space based on Equation (1) [19] to enable an effective colour-change detection. We determined the HSV range from which only the luminescence of the luminescent device was extracted, and the real-time-detected luminescent image was converted to the HSV colour space. We optimised the HSV range using SEL luminescence images before each experiment, allowing us to reliably detect luminescence.

(1) $$V\leftarrow \max\left(R,\;G,\;B\right)\;S\leftarrow \{\begin{array}{cc} \frac{V-\min\left(R,\;G,\;B\right)}{V} & if\;V\neq 0\;\\ 0 & otherwise \end{array}\;H\leftarrow \{\begin{array}{ccc} 60\left(G-B\right)/\left(V-\min\left(R,\;G,\;B\right)\right) & & if\;V=R\\ 120+60\left(B-R\right)/(\left(V-\min\left(R,\;G,\;B\right)\right) & & if\;V=G\\ 240+60\left(B-R\right)/(\left(V-\min\left(R,\;G,\;B\right)\right) & & if\;V=B\\ 0 & & \;\;\;\;\;\;\;\;\;if\;R=G=B \end{array}\;\mathrm{if}\;H<0,\;\mathrm{then}\;H\leftarrow H+360.\;\mathrm{On}\;\mathrm{output}\;0\leq V\leq 1,\;0\leq S\leq 1,\;0\leq H\leq 360.$$

### 2.2. Setup for Evaluating the Luminescent Devices

**Figure 1:**
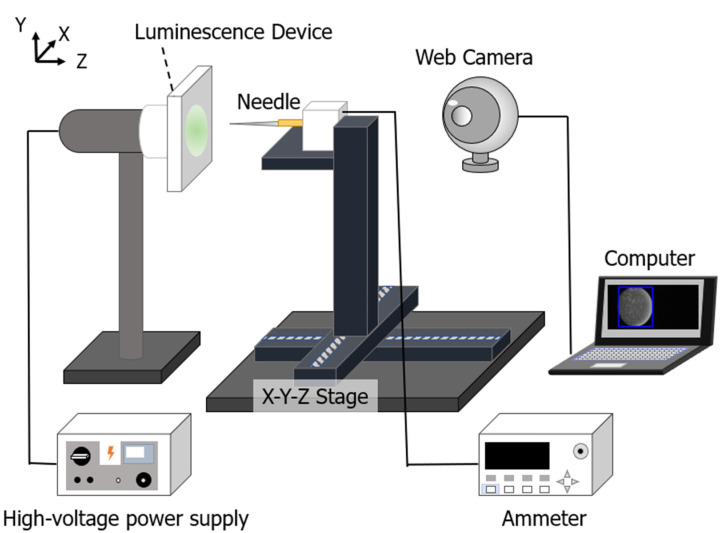
Experimental system for evaluating the prepared luminescence devices.

We constructed a system to apply a high voltage and measured the voltage and current using a needle (1 mm in diameter) instead of a finger to evaluate the prepared thin luminescent device (Figure 1). Here, the base substrate of the device used was aluminium foil. In this study, we used a high-voltage power supply (Green Techno Co., Ltd., GT100, Kawasaki, Japan) capable of applying voltages in the range from 0 to 40 kV, an automated stage (Sigma Koki Co., Ltd., OSMS26-200, Saitama, Japan) whose position can be controlled to be in the range 0–200 mm, an ammeter (ADC Co., Ltd., 5350, Saitama, Japan) for measuring minute currents, and a web camera (TC-PC8Z, I-O DATA Corporation, Kanazawa, Japan), which was used to capture the luminescence. The needle was placed on the X-Y-Z stage and connected to an ammeter (Figure 1). Using this system, we increased the distance between the thin luminescent device and the needle from 20 to 200 mm in increments of 20 mm and applied a voltage until the thin luminescent device emitted luminescence; the voltage and current were measured simultaneously.

SEL is emitted by the part of the thin luminescence device located at the shortest distance from a finger [14]. We assumed that the position at which a finger points to the thin luminescent device and the position of the SEL are approximately the same, and detected the position and measured its accuracy by analysing the images of the luminescence captured by a web camera. For these measurements, a circular thin luminescent device with a diameter of 60 mm was used. The distance between the needle and the luminescent device was fixed at 80 mm. When a voltage of 5 kV was applied to the luminescent device, the needle moved 40 times along the X-axis (horizontal to the ground) or Y-axis (vertical to the ground) by 1 mm and covered a total of 40 mm. The moving luminescence was captured using a web camera, and the position of the luminescence was measured by image processing.

### 2.3. Setup for Touchless Verification

**Figure 2:**
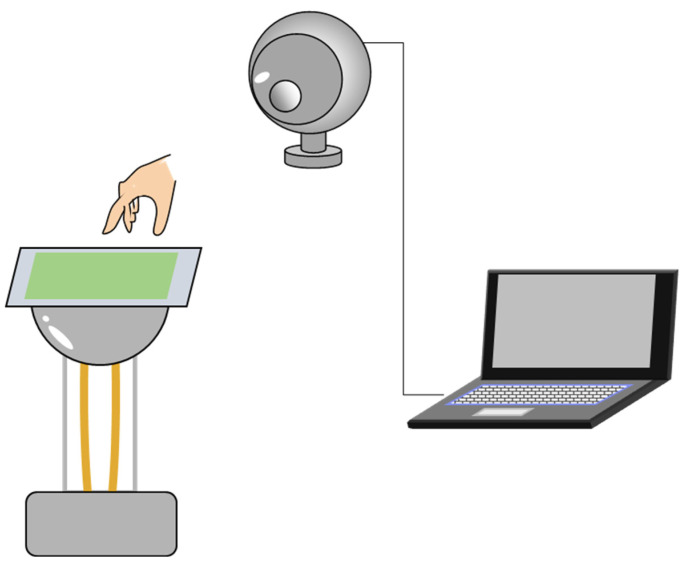
Experimental system for touchless verification.

To validate the applicability of the thin luminescent device as a touchless input device, we demonstrated real-time detection of the position of a moving luminescence following the movement of a finger. The principle of SEL assumes that light emission is induced by the interaction between a light-emitting material and an external charge, so light emission can occur even if the base material in this device is not metal. Here, the following experiment was conducted using paper as the base material. A luminescence device (200 mm × 200 mm) coated with epoxy resin and SrAl_2_O_4_:Eu^2+^ [14,15] on paper and a web camera were used (Figure 2) for this case. A Van de Graaff generator (Artec Corporation, 8951, Osaka, Japan) was used to apply a voltage (20 kV ± 1 kV) to the luminescent device, and the luminescence emitted by the device upon close proximity to a finger was captured using the web camera. The position coordinates corresponding to the centre of the luminescence area were calculated from the captured images and drawn as red circles in a window for the paint tool to visualise the movement of luminescence traced on a finger in real time.

## 3. Results

### 3.1. Relationship between Non-Contact Distance to Needle and Luminescence with Applied Voltage

**Figure 3:**
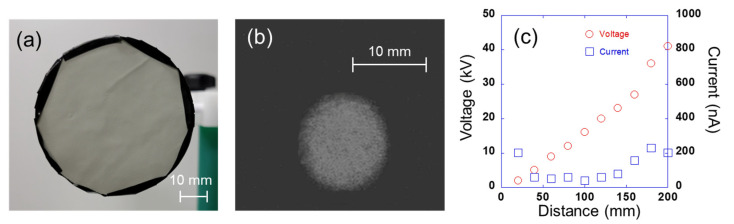
(**a**) Thin luminescent device, (**b**) example of luminescence, and (**c**) relationship between the voltage or current and the non-contact distance during the light emission by the prepared thin luminescent device.

The relationship between the electrical characteristics (voltage and current) observed during the luminescence was examined by varying the non-contact distance between the thin luminescent device (on which the voltage was applied) and the needle. Figure 3a,b show the luminescent device and the light emitted by it, respectively. Figure 3c presents the measured voltage and current when the non-contact distance from the needle was varied from 20 to 200 mm. Evidently, a longer non-contact distance between the needle and the light emitter results in a higher voltage during the light emission. For the thin luminescent device fabricated in this study, the correlation coefficient between the non-contact distance and the voltage is 0.98, and that between the non-contact distance and the current is 0.45. These results reveal a stronger correlation [20] between the voltage and current produced during the SEL emission. In addition, the current is approximately 250 nA or less, which is within the error range of this system.

**Figure 4:**
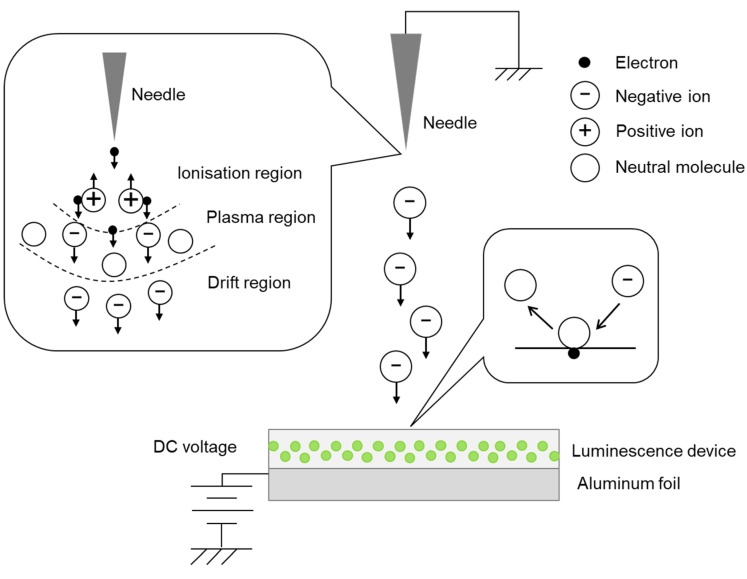
Schematic showing emission of negative ions from the needle electrode and charge transfer to/from the luminescent device.

The observed SEL phenomenon presumably originates from the movement of electric charges in air in the dark discharge region, which also includes corona discharges. The space between the two electrodes of a negative corona discharge can be divided into three regions, viz., the ionisation, plasma, and drift regions. The electrons near the needle electrode collide with neutral air molecules under a strong electric field at the beginning of the discharge. As a result, the neutral molecules are ionised into positive ions and electrons; the positive ions move towards the negative electrode under the applied electric field, whereas the electrons continue to ionise other neutral molecules. The electrons enter the plasma region after exiting the ionisation region. However, the electric field strength in the plasma region cannot provide sufficient energy to the electrons. Consequently, the electrons combine with neutral air molecules to form negative ions in the plasma region, enter the drift region, and travel towards the positive electrode [21]. In other words, in the region of dark discharge, including corona discharge, negative ions are emitted from the needle electrode, and these negative ions possibly enter the luminescent device because of the potential difference between the needle electrode and the ground (Figure 4). SrAl_2_O_4_:Eu^2+^ is a green phosphor, and it has been reported that there is an interdependent relationship between force, light, and electricity [22]. In addition, it has been confirmed that SrAl_2_O_4_:Eu^2+^ could emit intense green light when excited by lower direct current (DC) or alternating current (AC) voltages [23]. Therefore, SEL may be part of electroluminescence. The general charge injection mechanism for electroluminescence in an EL device follows several sequential steps [24]. When an applied voltage exceeds the threshold voltage, electrons are excited into the conduction band of the phosphor. They are then accelerated by the electric field, at which point they possess enough kinetic energy to excite a luminescent centre through impact, and a high-energy, “hot” electron is produced. The hot electrons then energetically relax by recombining with holes in the donor layer, through which a photon is emitted [25,26]. Such luminescence has been actively studied not only for electroluminescence but also for mechanoluminescence, and multiple mechanisms have been proposed [15,27,28,29,30,31]. Presently, the details of charge interactions during SEL are not known; however, it is hypothesized that the charges or ions act as external stimuli and induce luminescence through charge transfer.

### 3.2. Evaluation of Luminescence Position-Detection Accuracy

**Figure 5:**
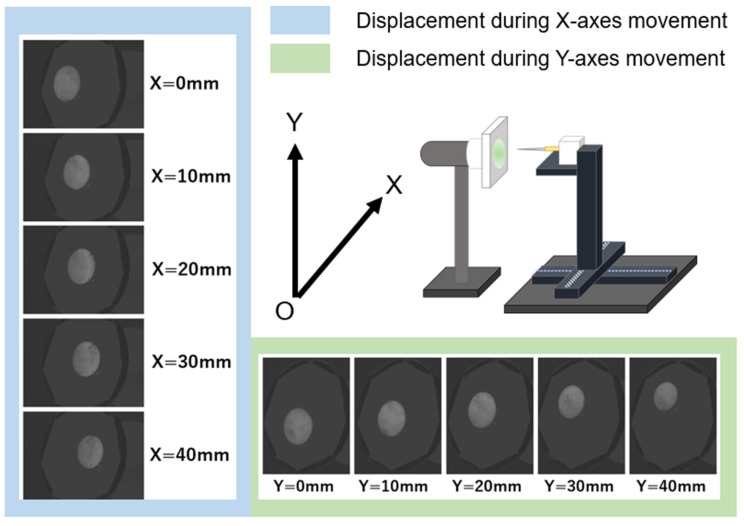
Displacement of luminescence position during the automatic stage movement.

**Figure 6:**
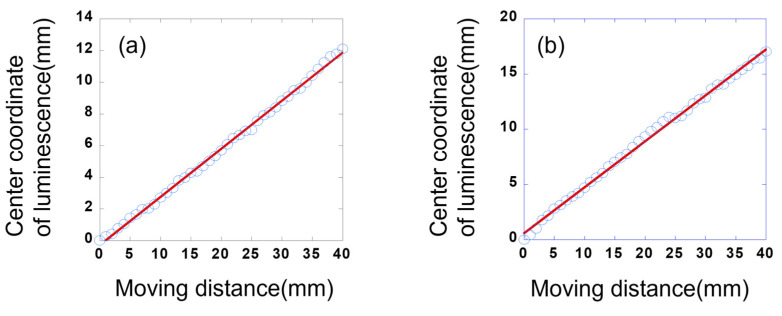
Relationship between the distance moved by the X-Y stage and the displacement of the central coordinates of the luminescence in the (**a**) X-axis and (**b**) Y-axis. Blue circles are correlation plots between centre coordinate position of luminescence and actual displacement. Red line is regression line.

Figure 5 depicts the relationship between the movement of the luminescence upon the movement of the automated stage and that of the needle in the X- or Y-axes. In Figure 5, the luminescence appears to move within the device at regular intervals on both the X- and Y-axes. The distances moved by the needle and the central coordinates of the luminescence along the X- and Y-axes are shown in Figure 6. Here, the centre coordinates of the luminescence were calculated by taking the average XY-axes coordinates of the upper left and lower right vertices of a rectangle that encloses the recognised luminescence outline. A simple regression analysis reveals a strong correlation (correlation coefficient: 0.99) between the distance travelled by the needle and the central coordinates of the luminescence on both the X- and Y-axes. As shown in Table 2, when the distance between the needle and the luminescent device is 80 mm, the maximum deviations from the regression line are 0.36 and 0.61 mm with respect to the X- and Y-axes, respectively. When the needle was moved 40 mm, the luminescence moved 12.11 mm along the X-axis and 18.09 mm along the Y-axis.

These results show that at a distance of 80 mm between the luminescent device and the needle, the position of the needle pointing to the luminescent device can be detected with an accuracy of less than 1 mm. However, the distance moved by the luminescence is smaller than that by the needle, and the distances moved on the X- and Y-axes are different. These differences are related to the size of the luminescent device and are possibly caused by the non-uniform electric field of the device. In detail, the observed difference between the distances moved along the X- and Y-axes is possibly caused by two reasons: First, the central point of the moving needle does not coincide with that of the luminescent device. Second, the effect of the non-uniform electric field may be different for the X- and Y-axes. In this experiment, we used a needle to enhance positional accuracy. As shown in Figure 3b, the diameter of the luminescent spot was about 10 mm. Varying the diameter or shape of the needle could reduce the size of the luminescent spot, and there is a possibility of reducing the error.

### 3.3. Demonstration of Touchless Technology Using the Fabricated Luminescent Devices

**Figure 7:**
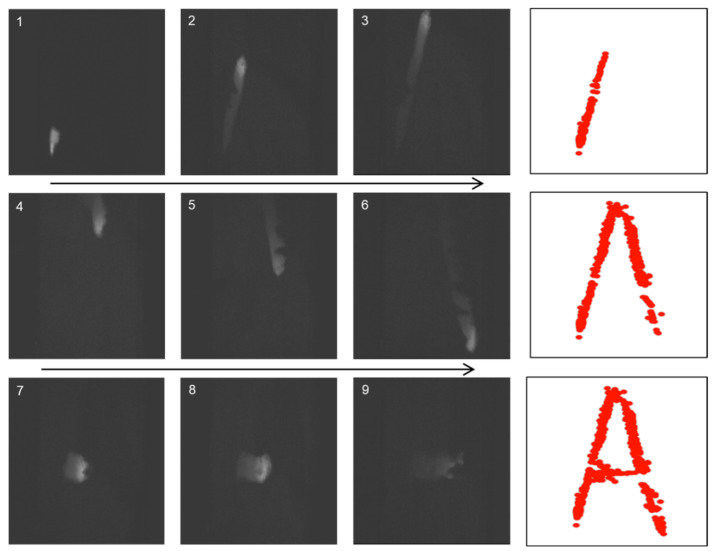
Results of interactive feedback with a paint tool based on the developed touchless technology. Numbers in the figure is state number of the procedure to write. Red dots indicate the position of the detected luminescence.

We employed the system shown in Figure 2 and used a web camera to capture the SEL emitted when a finger was in close proximity to the luminescent device; in this case, the finger traced an ‘A’ in the air. Figure 7 shows the detected luminescence, which is depicted by a series of red dots at the points of detection; the developed paint tool was used to indicate these red dots. The position of the luminescence was detected by software and a web camera installed behind the finger, and it was confirmed that information on the position of the person pointing at the device could be obtained in real time from the detected position of the luminescence emitted by the device. However, as shown in Figure 7 (photo 6), some areas do not show any luminescence, possibly because an insufficient amount of luminescent paint was applied to the sample. Although we can visualise the ‘A’ traced by the moving finger using the developed software, the thickness of the line is not constant, and the red dots do not form a clean straight line, possibly because a red circle of the same size is drawn at the central coordinate of the luminescence irrespective of the thickness of the luminescence. This result suggests that a clean straight line may be obtained by changing the size of the red circle depending on the luminescence thickness. Nevertheless, we succeeded in utilising SEL in the non-contact mode by capturing the movement of a finger in the form of luminescence using a web camera and treating it as an input signal.

## 4. Conclusions

In this study, a voltage-based luminescent device and support rod or finger were used to verify the distance, position-detection accuracy, and real-time performance of a light-emitting element with a simple structure. The light-emitting structure was fabricated by simply coating a light-emitting material on a substrate such as metal or paper, and subsequently, a voltage such as static electricity was applied to the fabricated device. Using this luminescent device, we confirmed that SEL occurred at a non-contact distance of at least 80 mm from the support rod. The detection of SEL enabled the detection of the position of the finger with a high spatial resolution of less than 1 mm using a low-cost web camera. This technology facilitates a non-proximal detection of a target object using finger movements (in the Z-axis) and provides highly accurate position information (XY-axes) as the input. Furthermore, we succeeded in establishing touchless technology based on a new principle by detecting SEL following the movement of a finger in real time. In the developed system, a range of specified hue, saturation, and value (HSV) is obtained from an image captured by a camera. Therefore, objects of complex geometric shapes can be recognized with high reproducibility. This luminescent device is easy to install, as it can be simply attached to a metal plate connected to a high-voltage power supply. The advantages and verification results of the developed device are expected to pave the way for the development of low-cost, easy-to-implement, and contactless devices.

## Figures and Tables

**Table 1:** Principles and features of touchless input interfaces.

	Retrofit to Wall	Space Constraint	Non-Proximity Sensor	Positional Accuracy	Figure
Camera (facing)	Bad	Good	Good	Good	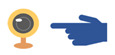
Camera (upper)	Good	Bad	Good	Neutral	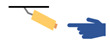
Ultrasonic	Bad	Good	Neutral	Neutral	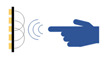
Infrared sensor	Neutral	Good	Bad	Good	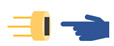
Touch panel	Good	Good	Bad	Good	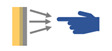

**Table 2:** (A) Maximum deviation from the regression line and (B) distance moved by the luminescence.

	X Coordinate (mm)	Y Coordinate (mm)
(A)	0.36	0.61
(B)	12.11	18.09

## Data Availability

Data sharing not applicable.
